# Misperception of body weight and associated socioeconomic and health-related factors among Korean female adults: A nationwide population-based study

**DOI:** 10.3389/fendo.2022.1007129

**Published:** 2022-12-23

**Authors:** Yoonjung Yoonie Joo, Jina Kim, Kiwon Lee, Geum Joon Cho, Kyong Wook Yi

**Affiliations:** ^1^Institute of Data Science, Korea University, Seoul, Republic of Korea; ^2^Department of Statistics, Korea University, Seoul, Republic of Korea; ^3^Department of Food and Resource Economics, College of Life Sciences and Biotechnology, Korea University, Seoul, Republic of Korea; ^4^Department of Obstetrics and Gynecology, College of Medicine, Korea University, Seoul, Republic of Korea

**Keywords:** perceived weight, weight misperception, weight management, health informatics, body mass index, public health

## Abstract

**Background:**

Misperception of body weight is associated with various psychological and health problems, including obesity, eating disorders, and mental problems. To date, female-specific risk factors, including socioeconomic or health-related lifestyle features, or their indicative performance for the misperception in Asian women according to age groups remain unknown.

**Objectives:**

To investigate the prevalence and associated risk factors for the mismatch in self-perceived body weight and evaluated the classification performance of the identified risk factors across age groups in female adults.

**Methods:**

We analyzed data of 22,121 women (age 19–97 years) from the 7-year Korea National Health and Nutrition Examination Survey dataset (2010-2016). We evaluated self-perceived body weight of the participants with their actual weight using the body mass index cut-off and grouped them by age: early adulthood (19–45), middle adulthood (46–59), and late adulthood (≥60). Logistic regression was conducted in each age group based on their weight misperception. The classification performance of the identified risk factors was evaluated with a bagging tree ensemble model with 5-fold cross-validation.

**Results:**

22.2% (n=4,916) of the study participants incorrectly perceived their body weight, of which 14.1% (n=3,110) and 8.2% (n=1,806) were in the underestimated and overestimated groups. Among the age groups, the proportion of participants who misperceived their body weight was highest in late adulthood (31.8%) and the rate of overestimation was highest in early adulthood (14.1%). We found that a lower education level, absence of menopause, perception of themselves as unhealthy, and efforts for weight management were significantly associated with the overall misperception (overestimation or underestimation) of body weight across age groups. Based on the identified risk factors, the highest area under the receiver operating curve (AUROC) and accuracy of the best classification model (weight overestimation in all participants) were 0.758 and 0.761, respectively. Adding various associated lifestyle factors to the baseline model resulted in an average increase of 0.159 and 0.135 in AUROC for classifying weight underestimation and overestimation, respectively.

**Conclusions:**

Age, education level, marital status, absence of menopause, amount of exercise, efforts for weight management (gain, loss, and maintenance), and self-perceived health status were significantly associated with the mismatch of body weight.

## 1 Introduction

Obesity is a complex and multifactorial disease that is a major public health issue. The prevalence of obesity has been epidemically increasing worldwide; being overweight and obese affects over one-third of the world’s population (1), and it is expected that 38% of the world’s adult population will be overweight, and 20% will be obese by 2030 (2). Excess weight and obesity are well-established critical risk factors for comorbidities of chronic diseases such as type 2 diabetes, hypertension, dyslipidemia, cardiovascular events, or premature mortality (3, 4). Obesity represents a challenge in public health given its high prevalence, increasing trend, and accompanying health risks (2).

Issues of obesity and its related health problems have generated an increased attention to the perception of and satisfaction with body image and shape. Body image distortion can lead to inappropriate diet restriction, eating disorders, and excessive weight loss, which in turn, give rise to reproductive function issues, such as menstrual irregularity, hypothalamic dysfunction, infertility, and bone loss due to impaired sex hormone metabolism (5–9). Moreover, body dissatisfaction is linked to adverse psychological consequences such as poor self-esteem, depression, and anxiety (10–12).

Existing studies report certain factors associated with the discrepancy between perceived and actual body shape and size (13–15). Significant gender and age differences have been reported for this discrepancy; males and younger individuals tend to underestimate their weight (and vice versa for females) (14–17). Studies have explored comprehensive lifestyle factors, ranging from health-related behaviors to socioeconomic status; however, most of this research used cross-sectional designs, highly selected cohorts, or populations with specific age groups (e.g., high school or university students, and young adults) (18–22). In addition, there is growing attention on how to identify the target populations with weight misperception, and only a few studies have performed predictive analytics based on the associated behavioral and social factors that lead to body weight misperception.

This study investigated the perceived body weight and/or its disturbance and trends across age groups in Korean women, using large population data from the national registry to determine the associated female-specific and age-specific risk factors for the discrepancy between actual body size and self-perceived body weight. One of the main purposes of the present study was to predict the future occurrence of body weight misperception with a machine-learning approach, by providing data that could be used by the public health branch management for preventive interventions.

## 2 Materials and methods

### 2.1 Study population

Data from 22,121 Korean women aged 19–97 years (mean age 50.4 ± 16.6) included in this study were collected from the Korea National Health and Nutrition Examination Survey (KNHANES) performed between 2010 and 2016. The KNHANES is a nationwide survey that has been conducted by the Division of Chronic Disease Surveillance under the Korea Centers for Disease Control and Prevention since 1998. This annual program evaluates health-related behaviors and nutritional status assessments for representative South Korean populations. Specialized research teams that consist of selected professional investigators, including nurses, nutritionists, and students majoring in public health after completing 1 month of training, undertake health assessments and perform conducted interviews and physical examinations with participants. The study was approved by the institutional review board of Korea University Ansan Hospital.

### 2.2 Sociodemographic and lifestyle variables

The KNHANES survey questionnaires assessed a wide range of lifestyle factors, ranging from health-related behaviors to socioeconomic status. Data on age, education level, income level, marital status, self-perceived health status, alcohol consumption, and employment status were obtained using structured survey items. Education level was categorized as elementary school or lower, middle school, high school, and college or higher. Income level was classified according to monthly income quartiles for each member in the household: low, middle-low, middle-high, and high. Participants consuming more than 12 alcohol drinks per year were classified as alcohol drinkers. The following clinical or health-related information were collected: past medical history of chronic diseases, experience of anxiety and depressive mood (none/mild/severe), degree of chronic pain (none/mild/severe), days of anaerobic workout per week, days of walking per week, exercise for weight loss, weight-control practice, gravidity, and menstruation condition. Past medical history of chronic diseases was defined as having been diagnosed from a physician, which was dichotomized into present and none for each disease, including diabetes, hypertension, hyperlipidemia, or depression. Weight-control practice was divided into the following categories: “weight loss management,” “weight maintenance management,” “weight gain management,” and “never tried to control weight”. Gravidity was defined as the number of pregnancies the woman has ever had, including all live births, stillbirths, miscarriage and abortions. The menstruation condition of the participants was assessed whether they were menstruating or were amenorrheic due to pregnancy, lactation, or menopause (menopause type). The full list of the variables used in this study is provided in **Supplementary Table 1**.

### 2.3 Assessment of objective weight status and classification of body weight perception

To assess the objective weight status, anthropometric indices, including height, weight, and waist circumference, were measured, and the body mass index (BMI) was calculated using the formula [bodyweight (kg)/(height x height) (m^2^)]. In addition, information on the self-perceived body image was collected based on the following questionnaire: “Do you consider yourself as (1) very thin, (2) a little thin, (3) average, (4) a little obese, or (5) very obese.” Based on a comparison between the subjects’ actual weight status (measured using BMI) by the World Health Organization Asian-Pacific criteria (23) and their self-perceived body image, two categories of mismatched body images were generated: (1) overestimation, defined as women who had normal weight or were underweight (BMI less than 23) who perceived their body as a little obese or very obese and (2) underestimation, defined as women with a BMI ≥ 23 (overweight or obese) who responded that their body size was average, a little thin, or very thin. The control group was defined as women with normal perception who were not belong to either of overestimation or underestimation categories.

### 2.4 Statistical analysis

First, Chi-square test was performed to examine linear trends in proportions of weight misperception among age groups. Then, we fitted multivariate logistic regression models to examine the associations between overall weight misperception and a range of lifestyle factors for all the participants. The main null hypothesis of our logistic regression model was that there is no association between specific lifestyle factors and weight misperception. Among the tested factors, gravidity was the only continuous variable and we replaced its extreme outliers (calculated as the 3rd quartile + 3 × the interquartile range or 1st quartile—3 × the interquartile range) with median values.

To explore age-specific differences in lifestyle factors associated with weight misperception, we classified the participants into three age groups and repeated the analysis as follows: early adulthood (19–45 years), middle adulthood (46–59 years), and late adulthood (≥ 60 years) (mean ages of 33.8, 52.6, and 69.8 years, respectively). For age-specific analysis, different subsets of health-related and lifestyle factors, including age, alcohol consumption, anxiety and depressive mood, chronic pain, days of anaerobic workout, days of walking per week, education level, exercise for weight loss, gravidity, income level, marital status, self-perceived health status, and management of weight (gain, loss or maintenance) were tested (**Supplementary Table 2**). The Bonferroni-adjusted significance level of 0.0055 for multiple comparisons (3 different age groups, each with overall misperception, overestimation, and underestimation patterns) was applied. All the analyses were performed in the R environment (version 4.1.1) using the *svyglm* function in the survey package, accounting for the KNHANES sampling weights.

### 2.5 Predictive analysis

We trained an ensemble of decision tree models to classify the participants’ self-perceived body weight status based on the identified lifestyle factors from the aforementioned regression analysis. We hypothesized that several behavioral and social lifestyle factors would account for the misperception of body weight. For the model training and evaluation, the data were split into training (70%) and test (30%) sets, among which the normal controls were randomly undersampled. The baseline model was constructed only including BMI, and its result was compared with our combined model that additionally incorporated significantly identified lifestyle factors. The model performance was evaluated using the held-out test set by computing the values of the area under the receiver operating characteristic curve (AUROC), the 95% confidence interval (CI), and accuracy.

## 3 Results

### 3.1 Demographic characteristics and overall misperception patterns of body weight

We analyzed multivariate data from 22,121 Korean women who participated in the KNHANES between 2010 and 2016. **Table 1** presents the demographic characteristics of the participants. A wide range of socioeconomic, lifestyle, and health-related factors were included in the analysis, ranging from self-reported questionnaire data, reporting the individual’s environment or daily behaviors, to objective health/weight measurements or clinical diagnosis history (**Supplementary Table 1**).

**Table 1:** Demographic characteristics of the study participants.

	Total n = 22,121		
	Early adulthood (age 19 - 45)(n = 9,006)	Middle adulthood (age 46 - 59)(n = 5,909)	Late adulthood (age ≥ 60)(n = 7,206)
Age	33.8 ± 7.4	52.6 ± 3.9	69.8 ± 6.5
BMI	22.4 ± 3.6	23.9 ± 3.3	24.4 ± 3.3
Gravidity	1.8 ± 1.7	3.6 ± 1.7	5.2 ± 2.4
Marital status			
married	6312 (70%)	5826 (98.6%)	7170 (99.5%)
not married	2694 (30%)	83 (3.4%)	36 (0.5%)
Employment status			
working	4904 (54.5%)	3523 (60.1%)	4987 (69.2%)
Not working	4102 (45.5%)	2386 (39.9%)	2219 (30.8%)
Education level			
elementary school and below	79 (0.9%)	1264 (21.4%)	5209 (72.3%)
middle school and below	204 (2.3%)	1200 (20.3%)	868 (12%)
high school and below	3824 (42.5%)	2354 (39.8%)	822 (11.4%)
university and above	4899 (54.4%)	1091 (18.5%)	307 (4.3%)
Menopause status			
no menopause	8902 (98.8%)	1959 (33.2%)	38 (0.5%)
artificial	85 (0.9%)	3239 (54.9%)	690 (9.6%)
natural	19 (0.2%)	701 (11.9%)	6478 (89.9%)
Self-perceived body weight			
correct	7437 (82.6%)	4857 (82.2%)	4911 (68.2%)
underestimated	303 (3.4%)	673 (11.4%)	2134 (29.6%)
overestimated	1266 (14.1%)	379 (6.4%)	161 (2.2%)
Self-perceived health status			
very good	418 (4.6%)	223 (3.8%)	200 (2.8%)
good	2816 (31.3%)	1364 (23.1%)	1213 (16.8%)
usual	4616 (51.3%)	3098 (52.4%)	3244 (45%)
bad	1074 (11.9%)	1041 (17.6%)	1754 (24.3%)
very bad	82 (0.9%)	183 (3.1%)	795 (11%)

Age, BMI, and Gravidity; mean ± SD.

**Figure 1:**
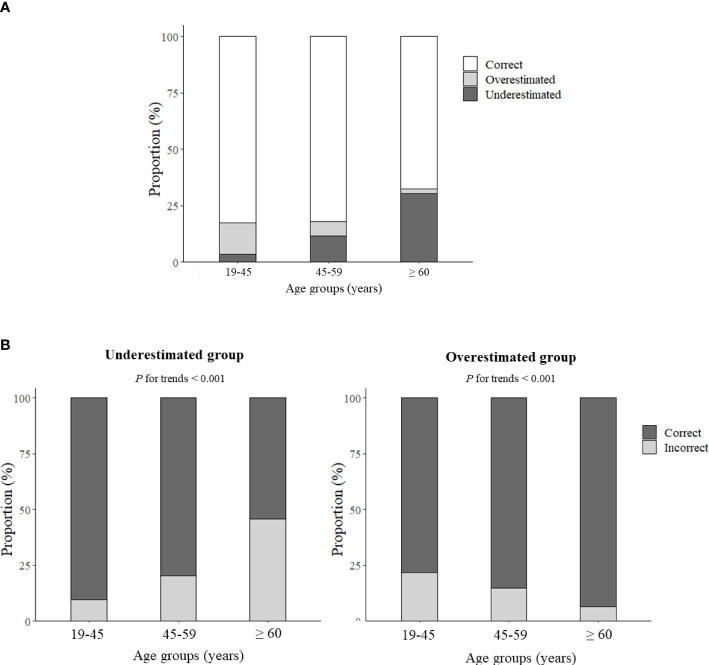
**(A)** Stacked bar graph showing the proportions of the self-perceived body weight status of Korean women by age group, and **(B)** the proportions of individuals with mismatched perception of body weight by age group.

The overall proportions for the “correct” and “mismatch” types of perceived body weight by age groups are shown in **Figure 1A**, where the age categories were 19–45 years (early adulthood), 46–59 years (middle adulthood), and ≥ 60 years (late adulthood). The proportion of participants who correctly perceived body weight was similar for early adulthood and middle adulthood (82.6% and 82.2%, respectively) but lower for the late adulthood group, as only 68.2% were correctly matched. Moreover, there was a trend towards an increase in the underestimation of weight with increasing age (*P* < 0.001); in contrast, the proportion of overestimation decreased with age (*P* < 0.001) (**Figure 1B**).

**Figure 2:**
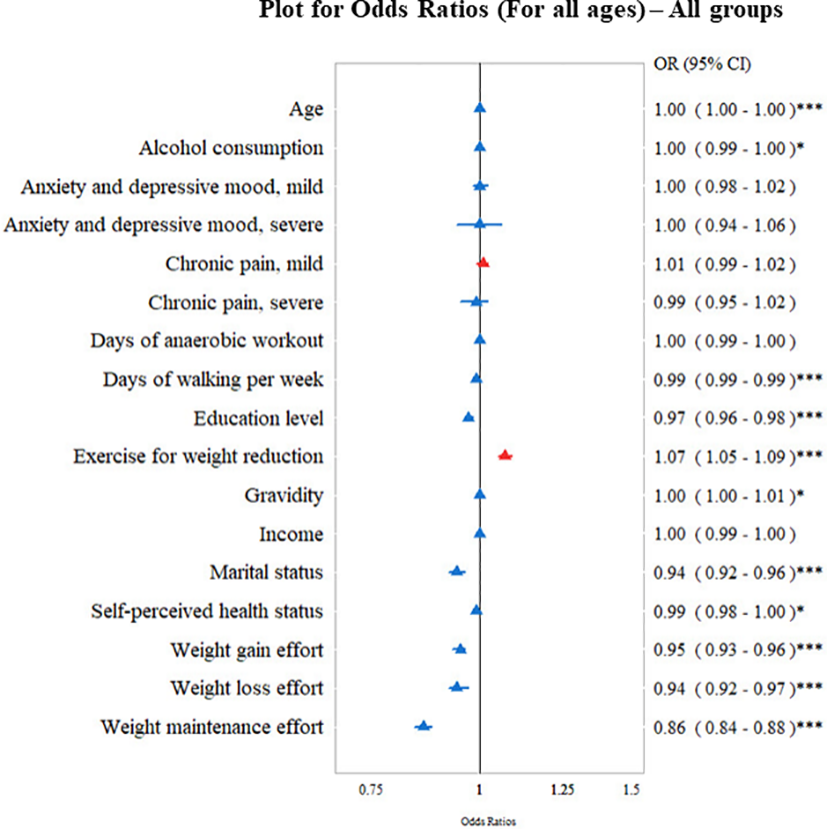
Study participants showing odds ratio (OR; 95% confidence interval [CI]) of socioeconomic and health-related factors associated with overall body weight misperception (either overestimation or underestimation). ^*^P < 0.05, ^***^P < 0.001.

We identified several lifestyle factors that were significantly associated with the overall misperception (overestimation and underestimation) of body weight (**Figure 2**). Age, alcohol consumption, days of walking, education level, marital status, self-perceived health status, effort of weight management (gain, loss, and maintenance) were negatively associated with misperception patterns, whereas, exercise behaviors for weight loss was positively associated with misperceived body weight.

### 3.2 Age-specific factors associated with misperception of body weight status

**Figure 3:**
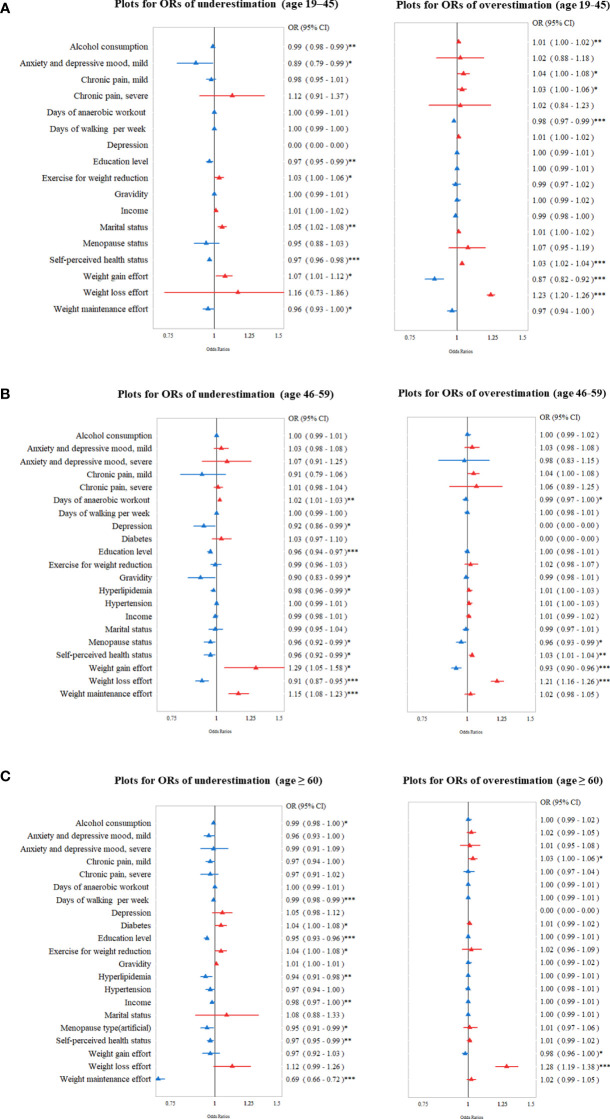
Odds ratio (OR; 95% confidence interval [CI]) of socioeconomic and health-related factors for body weight misperception (overestimation or underestimation) in different age groups: **(A)** 19–45 years **(B)** 46–59 years, and **(C)** ≥ 60 years. ^*^P < 0.05, ^**^P < 0.01, ^***^P < 0.001.

Based on the factors associated with misperception patterns, we analyzed the data using category-specific variables. In the early adulthood group (age 19 to 45), women who tended to consume less alcohol, had a lower education level, were married, and perceived themselves as unhealthy were significantly more likely to underestimate their body weight (**Figure 3A**, left). On the contrary, women who tended to consume more alcohol, had chronic pain, did fewer anaerobic workouts, perceived themselves as healthy, made less effort for weight gain and more effort for weight loss were more likely to overestimate their body weight (**Figure 3A**, right).

For the middle adulthood group (age 45–59 years), women who did more anaerobic workouts, had fewer episodes of depression, had a lower education level, had lower gravidity, and were not menopausal state were significantly more likely to underestimate the body weight (**Figure 3B**, left). Women who did less anaerobic workouts, were not in menopause, made less effort for weight gain and more effort for weight loss, and who perceived themselves healthier were significantly more likely to overestimate their body weight (**Figure 3B**, right).

In the late adulthood group (age ≥ 60 years), less alcohol consumption, fewer days of walking, lower education level, more exercise for weight loss, lower income, no menopause, and perceiving themselves as unhealthy were associated with an underestimation of body weight (**Figure 3C**, left). Among the variables of chronic medical diseases, the presence of diabetes and the absence of hyperlipidemia were significantly associated with an underestimation of body weight. Women who perceived themselves as healthier, with less effort for weight gain, and more effort for weight loss, were significantly associated with an overestimation of body weight (**Figure 3C**, right).

### 3.3 Predictive performance

**Figure 4:**
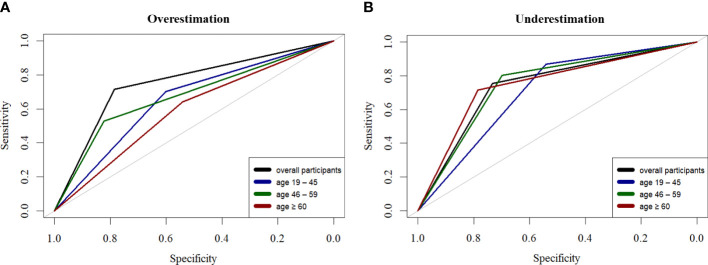
Performance of the decision tree-based ensemble model for predicting self-perceived body weight status in the overall dataset (n = 22,121); participants in the age groups of 19–45 years (n = 9,006), 46–59 years (n = 5,909), and ≥ 60 years (n = 7,206). **(A)** Receiver operating characteristic (ROC) curves of predicting overestimating pattern, and **(B)** underestimating pattern in different age groups.

The bagging tree ensemble model that included the previously identified lifestyle features showed a moderate overall performance for assessing weight overestimation (**Figure 4A**) and underestimation (**Figure 4B**). The feature included in each model were presented as footnote of **Supplementary Table 2**. Across the tested models, the highest AUROC and accuracy of the best classification model (weight overestimation in all participants across ages) were 0.758 and 0.761, respectively, with the 5-fold cross-validation (**Supplementary Table 2A**). Adding various lifestyle factors to the baseline model resulted in an average increase of 0.159 and 0.135 for AUROC when estimating weight-underestimating and weight-overestimating behavior, respectively. The classification models for the weight-overestimating behavior did not show any improvement compared to the baseline model, which only included BMI (**Supplementary Table 2B**). The feature important analysis revealed the most useful relevant variables for classifying weight underestimation, which were BMI, weight management status, and self-perceived health.

## 4 Discussion

In this study, we investigated the age-specific trend of self-perceived body weight in a large population of Korean women, and observed that the incidence of mismatch of body weight increases with age and is highest in late adulthood. Weight overestimation patterns were more commonly observed than weight underestimation in early adulthood (ages 19-45), and the pattern was reversed in middle adulthood (ages 45-59) and late adulthood (ages ≥ 60) in our study.

Existing studies have reported that race, gender, age, and behavioral factors influence the misperception of one’s body weight (16, 24–30). To date, most of these studies were conducted in the US or on Western populations, which have limited application to women in other countries (16). Unlike Western populations where an underestimation in overweight/obese women is the main issue, women in Asian countries tend to overestimate their body weight and attempt weight control more than Western women (31, 32). Our work represents one of the few studies examining the overall trend, prevalence, and age-specific patterns of body weight perception in Asian populations. Similar to the findings of other studies, we observed that young adults are likely to overestimate their body weight or image, and this phenomenon is frequently reported across countries and ethnicities (33–35). Young adults are often exposed to unrealistic body ideals portrayed in mass media that are regarded as the norm of societal standards of beauty (36). Accumulated studies have shown a high prevalence of misperception among the young population; however, data on older adults are currently limited. The results of the present study revealed that the proportion of individuals with a mismatch in body image was highest (31.8%) in the late adulthood group (≥ 60 years) in our cohort. Notably, underestimation (29.6%) was more prevalent than overestimation (2.2%) in this age group. A possible explanation for this finding is that age-related changes in overall cognitive function or reduced interest in one’s body image affect one’s perceived body image. The importance attributed to body image and physical appearance decreases with age, and this reduced emphasis on appearance may be protective against negative self-evaluations associated with body image (37). It is also possible that becoming feeble or care-dependent with age may cause the person to feel weak and misperceive themselves as underweight. More importantly, an awareness of this phenomenon of the misperception on body images is necessary because chronic diseases such as diabetes, hypertension, metabolic syndrome, and health issues related to obesity may be overlooked because older women do not feel the need for weight management (38).

In this study, we identified several sociocultural, habitual, health-related, and female-specific factors that were significantly associated with misperceptions of body weight in Korean women. Our regression analysis revealed that age, education level, days of walking, and efforts for weight management were negatively associated with a misperception of body weight. Of note, self-perceived health status was negatively related to the misperception of body weight across all age groups, indicating that women who perceived themselves as unhealthy tended to underestimate their body weight. Also, making specific efforts toward weight loss was associated with weight-overestimation patterns in all age groups; these results support earlier observations that dieters are more likely to overestimate their weights even though their weights were within a healthy range, and those bias could be risky as weight loss effort are a well-known risk factor for reduced nutritional state, mortality risk, or significant health problems (25, 39–41). It can also be suggested that dieters’ particular attention to their body weight are associated with biased estimates of weight status and this is likely to influence their subjective perception of healthiness, or the evaluation of ‘healthiness/unhealthiness’ might be closely related to one’s self-perceived weight status. Further work is required to clarify which of these two factors, weight loss efforts or self-perceived health status, is more important for the weight misperception behavior and if there is an interference or interaction between them.

One of the strengths of our study is that a large nationally representative sample was collected from a centralized dataset derived from a national registry cohort. Existing studies have reported that women tend to be more sensitive and overestimate their body weight than men (42–45). Our study aimed to demystify female-specific risk factors that might influence the weight misperception, including gravidity, menopausal status, or pregnancy history. In addition, we sought to identify women with weight misperceptions to quantify the extent of variance that the identified risk features could explain. To our knowledge, this is the first study with a machine learning approach to classify an individual’s weight misperception based on their demographic and lifestyle data. Even though we showed a slightly improved prediction model compared to the baseline model, we believe that the inclusion of more lifestyle and behavioral features will identify social determinants for weight misperception and could address some crucial public health concerns. By estimating the relative feature importance within the prediction models, we confirmed that an individual’s perceived health and weight management status were important and direct indicators of weight misperception.

Our study had several limitations. First, most of our lifestyle features were derived from self-reported questionnaire from the KNHANES dataset. Even though self-reported features enable deep curation of the environment surrounding an individual, they are inherently biased by an individual’s subjective feelings and do not comprehensively cover every part of the human lifestyle. Aside from the self-reported features, if other types and ranges of objective measurements were available to describe an individual’s lifestyle better, the findings would have been strengthened by identifying more reliable and valid risk features associated with weight misperception. In addition, we observed a significant case-control imbalance in our dataset, especially for weight underestimation, hindering the performance of our weight misperception prediction models based on a machine-learning approach. We attempted to address these challenges by undersampling the training data; however, for future studies, the use of a large population will strengthen the classification models and unveil meaningful outcomes. Lastly, even though the BMI is the most common anthropometric index to estimate general adiposity, it does not provide accurate information for distinguishing between fat and lean mass or fat distribution (46, 47). Thus, the use of BMI index alone might be insufficient to determine whether or not an individual is at her proper weights. Future studies should consider incorporating other anthropometric indices that can better identify the adiposity and fat distribution, to validate our findings further.

## 5 Conclusion

Our study identified several sociocultural, habitual, health-related, age-specific and female-specific factors that are significantly associated with misperceptions of self-perceived body weight in a large sample of Korean female adults. Our prediction models revealed that an individual’s perceived health and weight management status are important and direct indicators of weight misperception.

## Data availability statement

The raw data supporting the conclusions of this article will be made available by the authors, without undue reservation.

## Ethics statement

The studies involving human participants were reviewed and approved by Institutional Review Board, Korea University Ansan Hospital. Written informed consent for participation was not required for this study in accordance with the national legislation and the institutional requirements.

## Author contributions

YYJ, GJC, and KWY contributed to the conceptualization, design of the study, acquisition of the data, and writing of the original draft. YYJ, JK, and KL organized the database and performed the statistical analysis. All authors have contributed to the crucial discussion and manuscript revision and approved the submitted version.
