# Supplementary material for: Misperception of body weight and associated socioeconomic and health-related factors among Korean female adults: A nationwide population-based study
Source: Front Endocrinol (Lausanne). 2022 Dec 23;13:1007129. doi: 10.3389/fendo.2022.1007129 (PMC9816400; doi:10.3389/fendo.2022.1007129)
Supplement: Supplementary file 1 [file Table_1.docx]

**Supplementary Table 1. The list of variables included in this study.**

| **Factors and answer category** | **Description** |
| --- | --- |
| Age | Age of the respondents |
| BMI | BMI of the respondents |
| Marital status | Whether the respondent is single, married, or others (divorced, separated, or widowed) |
| Employment status | Whether the respondent is engaged to work for an employer (or self-employed) |
| Education level | Elementary school or lower, middle-school, high school, college and higher |
| Income | Personal income level of the respondents. |
| Menopause status | Self-reported menopausal status of the respondent, defined as having no period in the previous 12 months |
| Menopause type | Natural or surgical (artificial) menopause |
| Alcohol consumption | Frequency of alcohol consumption per year (days) |
| Self-perceived health status | Self-rated health status of the respondent. Very good (1), good (2), fair (3), bad (4), very bad (5). |
| Exercise to lose weight | Whether the participant exercised to lose weight over the past year |
| Chronic pain level | Presented by the respondent (none/mild/severe) |
| Anxiety and depressive mood | Presented by the respondent (none/mild/severe) |
| Gravidity | Number of pregnancies the woman has ever had, including all live births, stillbirths, miscarriages and abortions. |
| Hypertension | Whether the participant is ever diagnosed with hypertension Yes/No |
| Hyperlipidemia | Whether the participant is ever diagnosed with hyperlipidemia Yes/No |
| Diabetes | Whether the participant is ever diagnosed with diabetes Yes/No |
| Depression | Whether the participant is ever diagnosed with depression Yes/No |
| Weight management | Whether the respondent makes an attempt at weight control |
| Days of walking per week | Average number of days a week the respondent takes a walk |
| Days of anaerobic workout per week | Average number of days a week the respondent does anaerobic workout |

Income level for each age group were categorized as follows (unit: 10,000 Korean won):

- Early adulthood (19-45 years): lowest quantile (<136.53), middle-low quantile (136.53-206.99), middle-

high quantile (206.99-292.43), high quantile (293.43<)

- Middle adulthood (46-59 years): lowest quantile (<124.38), middle-low quantile (124.38-202.19),

middle-high quantile (202.19-311.47), high quantile (311.47<)

- Late adulthood (≥60 years): lowest quantile (<48.93), middle-low quantile (48.93-86.31), middle-high

quantile (86.31-161.15), high quantile (161.15<)
