# Supplementary material for: Misperception of body weight and associated socioeconomic and health-related factors among Korean female adults: A nationwide population-based study
Source: Front Endocrinol (Lausanne). 2022 Dec 23;13:1007129. doi: 10.3389/fendo.2022.1007129 (PMC9816400; doi:10.3389/fendo.2022.1007129)
Supplement: Supplementary file 2 [file Table_2.docx]

**Supplementary Table 2. Performance of prediction model.**

**(A) Performance of our integrative prediction model with 5-fold cross validation (including BMI and significantly identified lifestyle factors)**

|  | **Underestimated** | | | | | **Overestimated** | | | | |
| --- | --- | --- | --- | --- | --- | --- | --- | --- | --- | --- |
|  | **accuracy** | **precision** | **recall** | **f1** | **auc** | **accuracy** | **precision** | **recall** | **f1** | **auc** |
| overall participants | 0.753 | 0.729 | 0.755 | 0.742 | 0.743 | 0.761 | 0.751 | 0.780 | 0.765 | 0.758 |
| age 19-45 | 0.678 | 0.622 | 0.867 | 0.723 | 0.705 | 0.668 | 0.686 | 0.623 | 0.653 | 0.663 |
| age 46-59 | 0.730 | 0.756 | 0.800 | 0.771 | 0.750 | 0.729 | 0.632 | 0.753 | 0.687 | 0.675 |
| age ≥ 60 | 0.746 | 0.737 | 0.765 | 0.750 | 0.750 | 0.647 | 0.695 | 0.457 | 0.551 | 0.599 |

**(B) Performance of the baseline model that included only BMI**

|  | **Underestimated** | | | | | **Overestimated** | | | | |
| --- | --- | --- | --- | --- | --- | --- | --- | --- | --- | --- |
|  | **accuracy** | **precision** | **recall** | **f1** | **auc** | **accuracy** | **precision** | **recall** | **f1** | **auc** |
| overall participants | 0.721 | 0.756 | 0.718 | 0.736 | 0.593 | 0.609 | 0.644 | 0.663 | 0.653 | 0.527 |
| age 19-45 | 0.680 | 0.688 | 0.764 | 0.724 | 0.502 | 0.670 | 0.624 | 0.625 | 0.625 | 0.609 |
| age 46-59 | 0.703 | 0.708 | 0.789 | 0.746 | 0.525 | 0.627 | 0.637 | 0.615 | 0.625 | 0.518 |
| age ≥ 60 | 0.690 | 0.716 | 0.564 | 0.631 | 0.690 | 0.673 | 0.716 | 0.564 | 0.631 | 0.499 |

**- Input features included in the classification of overall participants:** Age, Alcohol consumption, Anxiety and depressive mood, Chronic pain, Days of anaerobic workout, Days of walking per week, Education level, Exercise for weight reduction, Gravidity, Income, Marital status, Self-perceived health status, Weight management effort

**- Input features included in the classification of participants with age 19-45:** Alcohol consumption, Anxiety and depressive mood, Chronic pain, Days of anaerobic workout, Days of walking per week, Depression, Education level, Exercise for weight reduction, Gravidity, Income, Marital status, Menopause status, Self-perceived health status, Weight management effort

**- Input features included in the classification of participants with age 46-59:** Alcohol consumption, Anxiety and depressive mood, Chronic pain, Days of anaerobic workout, Days of walking per week, Depression, Diabetes, Education level, Exercise for weight reduction, Gravidity, Hyperlipidemia, Hypertension, Income, Marital status, Menopause status, Self-perceived health status, Weight management effort

**- Input features included in the classification of participants with age≥60:** Alcohol consumption,

Anxiety and depressive mood, BMI, Chronic pain, Days of anaerobic workout, Days of walking per week,

Depression, Diabetes, Education level, Exercise for weight reduction, Gravidity, Hyperlipidemia, Hypertension,

Income, Marital status, Menopause type, Self-perceived health status, Weight management effort
